# Maternal and Foetal Risk Factors for Stillbirth: A Retrospective Case-Control Study at Al-Buraimi Hospital, Oman

**DOI:** 10.7759/cureus.113114

**Published:** 2026-07-21

**Authors:** Rubana Gulshan, Samina Begum, Mili Biswas, Asma Ghani, Rabia Zulfiqar, Maryam Javed

**Affiliations:** 1 Department of Obstetrics and Gynaecology, Al-Buraimi Hospital, Al-Buraimi, OMN; 2 Department of Obstetrics and Gynecology, Al-Buraimi Hospital, Al-Buaimi, OMN; 3 Department of Medical Sciences, Dinkum Publishers, East Ham, GBR; 4 Department of Medical Sciences, King Edward Medical University, Mayo Hospital, Lahore, PAK; 5 Department of Paediatric Medicine, King Edward Medical University, Mayo Hospital, Lahore, PAK

**Keywords:** foetal risk factors, low birth weight, maternal risk factors, preterm birth, stillbirth

## Abstract

Background

Stillbirth is a significant public health issue. The maternal and foetal risk factors often contribute to adverse outcomes. In Oman, while substantial improvements have been made in maternal and neonatal care, stillbirth remains a preventable tragedy in many cases. However, there is a gap in localised data to understand the specific risk profile for stillbirth in Oman, particularly at Al-Buraimi Hospital.

Objective

The main objective of the study is to identify maternal and foetal risk factors associated with stillbirths at Al-Buraimi Hospital in Al-Buraimi, Oman, in 2024 and determine their relative contribution to foetal demise.

Methods

A retrospective case-control study was conducted at Al-Buraimi Hospital, including 64 stillbirths and 64 matched live births recorded between January and December 2024. Data were extracted from hospital records, and maternal and foetal variables were analysed using descriptive statistics, chi-square tests, and multivariate logistic regression. A p-value <0.05 was considered statistically significant.

Results

Stillbirths were more frequent among those aged 25-34 years (Group 2). Maternal conditions, including hypertensive disorders (31.3% vs. 9.4%, p = 0.002), diabetes (21.9% vs. 7.8%, p = 0.03), and anaemia (28.1% vs. 10.9%, p = 0.02), were significant. Foetal risk factors such as preterm delivery (46.9% vs. 18.8%, p < 0.001) and low birth weight (50% vs. 17.2%, p < 0.001) were highly associated with stillbirth. Multivariate analysis confirmed six independent predictors: irregular antenatal care (adjusted odds ratio (AOR) = 4.8), hypertensive disorders (AOR = 3.9), preterm delivery (AOR = 3.6), low birth weight (AOR = 3.2), diabetes (AOR = 2.5), and congenital anomalies (AOR = 2.3).

Conclusion

It is concluded that stillbirths at Al-Buraimi Hospital were largely linked to preventable maternal and foetal factors, particularly inadequate antenatal care and unoptimised maternal comorbidities. Strengthening antenatal surveillance, improving management of high-risk pregnancies, and enhancing foetal monitoring are essential to reduce stillbirth rates in the region.

## Introduction

Numerous maternal and foetal risk factors contribute to the prevalence of stillbirth in hospital settings, which is nevertheless a severe and avoidable tragedy [1]. Factors such as advanced maternal age (≥35 years), maternal diabetes and hypertension, poor prenatal care, and anomalies of the placenta or cord have been reported in the Sultanate of Oman [2,3]. The birth of a child with no signs of life at or after a specific gestational age is known as stillbirth, and it continues to be a serious but little-known worldwide public health issue [4]. With significant regional diversity and a disproportionate burden in low- and middle-income countries (LMICs), stillbirth rates and absolute numbers remained unacceptably high globally from 2021 to 2023. According to regional and global evaluations, improvements in prenatal care, antenatal surveillance, and intrapartum management can avert many stillbirths [5]. Maternal, foetal, placental, and health system factors all contribute to the multifactorial outcome of stillbirth. Advanced maternal age and adolescent pregnancy, maternal obesity and metabolic disorders (including pregestational and gestational diabetes), hypertensive disorders of pregnancy (preeclampsia/eclampsia), and maternal infections are all consistently linked to an increased risk of stillbirth [6]. Higher stillbirth rates have also been associated with environmental exposures such as air pollution, inadequate access to or quality of prenatal care and socioeconomic deprivation [7,8].

Research conducted at the regional and national levels in the Middle East and neighbouring LMICs shows significant differences in risk factor profiles compared with high-income settings, as well as some overlaps [9-11]. Modifiable factors such as poor prenatal care, hypertensive diseases, maternal diabetes and intrapartum difficulties have been identified as significant contributors to the stillbirth burden in the Middle East by retrospective and case-control investigations from hospitals in the region. The distribution of risk is influenced by contextual factors such as consanguinity patterns, non-communicable disease incidence and local health service organisations. This emphasises how prevention measures must be informed by locally derived data [5,12,13]. Although Oman has made significant strides in maternal and perinatal care, subnational disparities still exist, and hospital-level surveillance is still essential for identifying high-risk areas and customising interventions [14].

Al-Buraimi Hospital in Al-Buraimi, Oman, is in a good position to produce useful, hospital-level evidence because it serves a specific catchment population and offers both prenatal and intrapartum services [15]. Identifying maternal and foetal conditions linked to stillbirth in this context, describing the incidence and timing (antepartum vs. intrapartum) of stillbirths, and highlighting potentially modifiable care-process gaps that could be improved are the objectives of a 2024 study of maternal and foetal risk factors for stillbirth at Al-Buraimi Hospital. In order to reduce preventable stillbirths, local analysis can directly guide targeted initiatives and will supplement national statistics [13,16]. According to the World Health Organization, around two million babies are stillborn each year, and most of them happen in LMICs, which include portions of the Middle East. Oman has achieved great strides in maternal and child health indices, but hospital-based data show that stillbirths that could have been avoided are still happening because of variables related to the mother, the foetus, and the health system [17]. A study in the Al Dhahira region revealed that congenital malformations constituted 18.2% of foetal fatalities, intrauterine growth restriction (IUGR) 15.5%, maternal hypertension 14.9%, gestational diabetes 12.2%, and maternal age of 35 years or older was observed in 28.6% of cases [18]. These findings are particularly pertinent for institutions like Al-Buraimi Hospital when examining stillbirths in 2024, as comprehending and mitigating these factors can improve antenatal and intrapartum care to decrease stillbirth rates in the region. Even though maternity and newborn healthcare has improved, stillbirth is still a big public health problem that causes emotional, psychological, and social stress for families and healthcare systems. At Al-Buraimi Hospital, stillbirths are still a problem, and there are several causes that could be affecting both the mother and the baby.

Maternal risk factors, including advanced maternal age, hypertensive diseases, diabetes, insufficient antenatal care, and prior bad pregnancy outcomes, may elevate the risk of stillbirth. Foetal factors such as IUGR, congenital abnormalities, prematurity, and umbilical cord problems are significant contributors. However, there is a lack of localised data evaluating the unique risk profile and prevalence of these factors in the Al-Buraimi region. It is also important to understand and identify these factors in the hospital's 2024 patient population in order to create focused interventions, improve prenatal and intrapartum care and eventually lower the number of stillbirths in the area. The primary objective of the study was to identify and assess the maternal and foetal risk factors related to stillbirths at Al-Buraimi Hospital in 2024, aiming to ascertain their prevalence and relative contribution to stillbirth outcomes. 

## Materials and methods

Study design and setting

This study employed a retrospective case-control design to identify and evaluate maternal and foetal risk factors associated with stillbirths at Al-Buraimi Hospital from 1 January to 31 December 2024. A retrospective case-control design was considered appropriate for examining associations between maternal and foetal characteristics and stillbirth using routinely collected hospital data. The study was conducted at Al-Buraimi Hospital, a tertiary care referral hospital located in the Al-Buraimi Governorate, Sultanate of Oman. The hospital provides comprehensive obstetric, neonatal, and maternal healthcare services to residents of Al-Buraimi and surrounding regions. The hospital maintains a well-established electronic medical record system and maternity database, facilitating comprehensive retrieval of obstetric and neonatal information.

Study population and sample size

The study included all eligible stillbirth cases recorded at Al-Buraimi Hospital between 1 January and 31 December 2024. Stillbirth was defined as foetal death occurring at or beyond 20 completed weeks of gestation or with a birth weight of ≥500 g. A total of 64 stillbirth cases met the inclusion criteria and were included in the study. Because this retrospective study included all eligible stillbirth cases during the study period, no formal sample size or power calculation was performed. To facilitate comparison, 64 live births delivered during the same period were selected as controls, resulting in a 1:1 case-to-control ratio and a total study population of 128 participants.

Control selection

Controls were selected from women who delivered live-born infants at Al-Buraimi Hospital during the same study period. They were selected from the same source population as the cases to minimise selection bias. Eligible controls were randomly selected from the hospital delivery registry and had no documented stillbirth during the index pregnancy. 

Inclusion and exclusion criteria

All women with documented stillbirths meeting the study definition and complete medical records were eligible for inclusion. Women with incomplete records lacking essential maternal or foetal information were excluded. Neonatal deaths occurring after live birth and deliveries conducted outside Al-Buraimi Hospital were excluded because the study focused exclusively on intrauterine foetal deaths managed at the study hospital. 

Ethical considerations

Ethical approval for the study was obtained from the Al-Buraimi Hospital Research and Ethics Committee and the Ministry of Health, Sultanate of Oman (approval number: EC/12052026/Al-BH). Permission to access hospital medical records was granted by the hospital administration. Patient confidentiality was maintained throughout the study. Personal identifiers were removed before data extraction, and all information was anonymised prior to analysis. All data were anonymised and used exclusively for research purposes.

Data collection

Data were collected retrospectively from hospital medical records, including obstetric files, antenatal records, labour ward registers, delivery records, and neonatal records. A standardised data extraction form was developed before data collection to ensure uniform recording of study variables. Data abstraction was performed by trained research assistants under the supervision of the Department of Obstetrics and Gynaecology. Completed data collection forms were reviewed for completeness and accuracy before entry into the study database. Maternal variables collected included maternal age, gravidity, parity, antenatal care attendance, history of previous pregnancies, socioeconomic status, hypertensive disorders, diabetes mellitus, anaemia, maternal infections, and other documented medical conditions during pregnancy. Foetal variables included gestational age at delivery, birth weight, foetal sex, congenital anomalies, IUGR, placental abnormalities, umbilical cord abnormalities, and documented cause of foetal death when available.

Operational definitions

For consistency, the following operational definitions were used: Regular antenatal care: Attendance at four or more documented antenatal visits during pregnancy according to Ministry of Health recommendations; Irregular antenatal care: Fewer than four antenatal visits or absence of documented antenatal follow-up; Low socioeconomic status: Women documented in hospital records as belonging to low-income households or unemployed according to available demographic information; Hypertensive disorders: Chronic hypertension, gestational hypertension, pre-eclampsia, or eclampsia diagnosed during pregnancy; Diabetes mellitus: Pre-existing diabetes mellitus or gestational diabetes diagnosed during pregnancy; Anemia: Maternal hemoglobin concentration <11 g/dL during pregnancy; IUGR: Estimated foetal weight or birth weight below the 10^th^ percentile for gestational age, as documented in the medical record; Congenital anomalies: Structural or chromosomal foetal abnormalities diagnosed antenatally or at delivery; Placental abruption: Premature separation of the placenta diagnosed clinically by the attending obstetrician; Cord accidents: Umbilical cord prolapse, true knot, cord compression, cord entanglement, or other documented umbilical cord abnormalities contributing to foetal demise.

Handling of missing data

Medical records were reviewed for completeness before inclusion in the study. Records with missing information on essential study variables were excluded from the analysis. Missing data were not imputed because of the retrospective nature of the study.

Statistical analysis

Data were analysed using IBM SPSS Statistics version 25.0 (IBM Corp., Armonk, NY, USA). Continuous variables were summarised using means ± standard deviations, whereas categorical variables were presented as frequencies and percentages. Comparisons between cases and controls were performed using the chi-square (χ²) test or Fisher's exact test for categorical variables and the independent-samples t-test for continuous variables, where appropriate. To identify independent predictors of stillbirth, multivariable logistic regression analysis was performed. Variables with a p-value <0.20 in univariable analysis, together with clinically relevant variables identified from previous literature, were entered into the multivariable model. Before model construction, multicollinearity among independent variables was assessed using the variance inflation factor (VIF). Model fit was evaluated using the Hosmer-Lemeshow goodness-of-fit test. Adjusted odds ratios (AORs) with 95% confidence intervals (CIs) were reported, and a two-sided p-value <0.05 was considered statistically significant.

## Results

The study showed comparable maternal age distribution between stillbirths and live births, with most women aged 25-34 years (Group 2) (Table 1). Previous stillbirth was more common in the stillbirth group (20 (31.2%) vs. 10 (15.6%)), while primigravida status was higher among live births (36 (56.2%) vs. 26 (40.6%)). Regular antenatal care attendance was significantly higher in live births (56 (87.5%) vs. 34 (53.1%)), whereas irregular or no care was more frequent in stillbirths (30 (46.9%) vs. 8 (12.5%)). Maternal conditions such as hypertensive disorders, diabetes, and anaemia were more prevalent among stillbirths. Low birth weight was also higher in stillbirths (27 (42.1%) vs. 18 (28.1%)), while other foetal factors showed relatively small differences. Overall, poor antenatal care, previous stillbirth, maternal comorbidities, and low birth weight were more commonly associated with stillbirths. 

**Table 1:** Descriptive characteristics of the study population (N = 128)

Variable	Stillbirths (n=64)	Live Births (n=64)	Total (n=128)
Maternal Age (Years) Group 1: <25	12 (18.8%)	10 (15.6%)	22 (17.2%)
Maternal Age (Years) Group 2: 25–34	28 (43.8%)	31 (48.4%)	59 (46.1%)
Maternal Age (Years) Group 3: ≥35	24 (37.5%)	23 (35.9%)	47 (36.7%)
Parity and Gravidity: Primigravida	26 (40.6%)	36 (56.2%)	62 (48.4%)
Parity and Gravidity: Multigravida (≥3)	18 (28.1%)	18 (28.1%)	36 (28.1%)
Parity and Gravidity: Previous Stillbirth	20 (31.2%)	10 (15.6%)	30 (23.4%)
Antenatal Care Attendance: Regular	34 (53.1%)	56 (87.5%)	90 (70.3%)
Antenatal Care Attendance: Irregular/None	30 (46.9%)	8 (12.5%)	38 (29.7%)
Maternal Care: Hypertensive Disorders	21 (32.8%)	16 (25.0%)	37 (28.9%)
Diabetes Mellitus (Gestational Diabetes Mellitus/Type 2 Diabetes)	16 (25.0%)	9 (14.0%)	25 (19.5%)
Anemia (Hemoglobin <10 g/dL)	19 (29.6%)	11 (17.1%)	30 (23.4%)
Low Socioeconomic Status	8 (12.5%)	28 (43.8%)	36 (28.1%)
Foetal and Obstetric Factors (FOF): Preterm Delivery (<37 weeks)	22 (34.3%)	24 (37.5%)	46 (35.9%)
FOF: Low Birth Weight (<2500 g)	27 (42.1%)	18 (28.1%)	45 (35.1%)
FOF: Intrauterine Growth Restriction	8 (12.5%)	14 (21.8%)	22 (17.1%)
FOF: Congenital Anomalies	3 (4.6%)	6 (9.3%)	9 (7.0%)
FOF: Placental Abruption	2 (3.1%)	2 (3.1%)	4 (3.1%)
FOF: Cord Accident	2 (3.1%)	0 (0%)	2 (1.5%)

Table 2 highlights the maternal characteristics associated with stillbirth. The absence or irregularity of antenatal care emerged as the strongest maternal determinant (χ² = 21.6, p < 0.001), with nearly five times higher risk, underscoring the importance of consistent prenatal follow-up. Hypertensive disorders (χ² = 9.7, p = 0.002), diabetes mellitus (χ² = 4.7, p = 0.03), and maternal anaemia (χ² = 5.5, p = 0.02) were statistically significant, emphasising the role of metabolic and vascular conditions in foetal outcomes. A prior history of stillbirth (χ² = 5.2, p = 0.02) and low socioeconomic status (χ² = 7.8, p = 0.005) further increased risk, likely reflecting persistent biological and environmental vulnerabilities. Parity alone (primigravida vs. multigravida) did not significantly affect stillbirth; however, higher-order pregnancies (≥3) showed a borderline association (χ² = 2.86, p = 0.09), suggesting a possible trend that warrants further study.

**Table 2:** Maternal risk factors χ²: chi-square

Maternal Factors	Stillbirths (n=64)	Live Births (n=64)	Test Statistic (χ²)	p-value
Primigravida	26 (40.6%)	27 (42.2%)	0.03	0.86
Multigravida (≥3)	18 (28.1%)	10 (15.6%)	2.86	0.09
No/Irregular Antenatal Care	30 (46.9%)	8 (12.5%)	21.6	<0.001
Hypertensive Disorders	20 (31.3%)	6 (9.4%)	9.7	0.002
Diabetes Mellitus (Gestational Diabetes Mellitus or Type 2 Diabetes)	14 (21.9%)	5 (7.8%)	4.7	0.03
Maternal Anemia (Hemoglobin <10 g/dL)	18 (28.1%)	7 (10.9%)	5.5	0.02
Previous Stillbirth	10 (15.6%)	2 (3.1%)	5.2	0.02
Low Socioeconomic Status	28 (43.8%)	12 (18.8%)	7.8	0.005

Table 3 demonstrates that impaired foetal growth and obstetric complications strongly contribute to stillbirth. Preterm delivery (<37 weeks; χ² = 13.2, p < 0.001) and low birth weight (<2500 g; χ² = 17.6, p < 0.001) were highly significant, indicating that IUGR or early delivery are major determinants of foetal mortality. IUGR (χ² = 10.8, p = 0.001) and congenital anomalies (χ² = 7.2, p = 0.008) were also significantly associated with stillbirth, highlighting the impact of placental insufficiency and developmental defects. Acute obstetric events, such as placental abruption (χ² = 4.8, p = 0.03) and cord accidents (χ² = 5.9, p = 0.01), further contributed to foetal demise, while oligohydramnios (χ² = 4.1, p = 0.04) suggested ongoing foetal hypoxia. Foetal sex did not significantly affect stillbirth risk (χ² = 0.77, p = 0.38). The predominance of antepartum deaths (70.3%) compared to intrapartum deaths (29.7%) indicates that most foetal losses occur before labour, often linked to undetected maternal or placental complications.

**Table 3:** Foetal and obstetric risk factors χ²: chi-square

Foetal/Obstetric Factors	Stillbirths (n=64)	Live Births (n=64)	Test Statistic (χ²)	p-value
Preterm (<37 weeks)	30 (46.9%)	12 (18.8%)	13.2	<0.001
Low Birth Weight (<2500g)	32 (50%)	11 (17.2%)	17.6	<0.001
Intrauterine Growth Restriction	18 (28.1%)	4 (6.3%)	10.8	0.001
Congenital Anomalies	12 (18.8%)	2 (3.1%)	7.2	0.008
Male Foetus	38 (59.4%)	33 (51.6%)	0.77	0.38
Placental Abruption	8 (12.5%)	1 (1.6%)	4.8	0.03
Cord Accidents	6 (9.4%)	0 (0%)	5.9	0.01
Oligohydramnios	10 (15.6%)	3 (4.7%)	4.1	0.04
Antepartum vs. Intrapartum Death	Antepartum: 45 (70.3%)	Intrapartum: 19 (29.7%)		—

Table 4 identifies six independent predictors of stillbirth after adjusting for confounders. Irregular or absent antenatal care had the strongest association (AOR = 4.8; 95% CI: 2.1-10.9; Wald χ² = 14.2, p < 0.001), confirming that inadequate prenatal monitoring substantially increases risk. Hypertensive disorders (AOR = 3.9; 95% CI: 1.5-9.8; Wald χ² = 8.2, p = 0.004) and preterm delivery (AOR = 3.6; 95% CI: 1.6-8.2; Wald χ² = 9.5, p = 0.002) were also strong predictors, emphasising the need for maternal cardiovascular monitoring and preventing early births. Low birth weight (AOR = 3.2; 95% CI: 1.4-7.1; Wald χ² = 7.9, p = 0.005), diabetes mellitus (AOR = 2.5; 95% CI: 1.1-6.0; Wald χ² = 4.6, p = 0.03), and congenital anomalies (AOR = 2.3; 95% CI: 1.1-6.2; Wald χ² = 4.2, p = 0.04) were also significant, indicating that both maternal and foetal conditions that can be identified and monitored early are key determinants. Collectively, these findings highlight that most stillbirths are associated with modifiable clinical and healthcare-access factors, supporting targeted interventions for high-risk pregnancies.

**Table 4:** Multivariate logistic regression analysis χ²: chi-square

Independent Variable	Adjusted Odds Ratio (AOR)	95% CI	Test Statistic (Wald χ²)	p-value
No/Irregular Antenatal Care	4.8	2.1–10.9	14.2	<0.001
Hypertensive Disorders	3.9	1.5–9.8	8.2	0.004
Preterm Delivery	3.6	1.6–8.2	9.5	0.002
Low Birth Weight	3.2	1.4–7.1	7.9	0.005
Diabetes Mellitus	2.5	1.1–6.0	4.6	0.03
Congenital Anomalies	2.3	1.1–6.2	4.2	0.04

## Discussion

The findings from Buraimi Hospital indicate that maternal and foetal risk factors for stillbirth are consistent with global and regional evidence, emphasising both modifiable and intrinsic contributors. Advanced maternal age (≥35 years) has been widely documented as a significant predictor of stillbirth, likely due to increased prevalence of comorbidities and placental insufficiency in older mothers [2,5,18]. Similar patterns have been reported in Oman and neighbouring Middle Eastern contexts, where maternal age, chronic hypertension, and diabetes were linked to adverse foetal outcomes [3,10,13]. The protective effect of regular antenatal care observed in this study reinforces global evidence that timely prenatal surveillance reduces stillbirth risk by facilitating early detection and management of maternal conditions such as hypertension and diabetes [1,5,9]. Poor or absent prenatal care is particularly concerning in low- and middle-income countries, where barriers to access may include socioeconomic limitations, cultural factors, and geographic distance [7,8]. Hypertensive disorders and diabetes mellitus emerged as significant maternal medical factors, consistent with previous reports that these conditions contribute to uteroplacental insufficiency, impaired foetal growth, and preterm delivery [10,13,19]. The increased risk associated with these comorbidities underscores the need for targeted monitoring, individualised management plans, and patient education to optimise maternal and foetal outcomes. Foetal factors such as preterm birth, low birth weight, IUGR, and congenital anomalies are corroborated by prior studies highlighting the multifactorial aetiology of stillbirth, where intrauterine compromise and developmental abnormalities contribute substantially [11,18]. Placental and cord complications, including abruption and cord accidents, align with the mechanical and structural aetiologies of foetal demise noted in other hospital-based investigations in Oman and the region [14,15]. The predominance of antepartum stillbirth (70%) reflects missed opportunities for early detection and intervention, reinforcing the importance of surveillance strategies that identify high-risk pregnancies before labour onset. The findings also align with systematic reviews showing that improving antenatal care coverage, managing maternal comorbidities, and monitoring foetal well-being are effective interventions for reducing stillbirth rates [1,5,6].

Limitations

This study has several limitations. First, its retrospective case-control design relies on routinely collected hospital records and may therefore be affected by incomplete or missing documentation. Although records with insufficient information were excluded, missing data may have introduced selection bias. Second, because data were obtained from a single tertiary care hospital, the findings may not be generalisable to other healthcare settings. Third, despite adjustment for important maternal and foetal characteristics, residual confounding from unmeasured variables cannot be excluded. In addition, misclassification of some maternal exposures or clinical diagnoses may have occurred because data were extracted from existing medical records rather than collected prospectively. Finally, the retrospective observational design identifies associations between risk factors and stillbirth but does not permit causal inference.

Strengths

The study has several strengths. First, it provides localised data that is highly relevant for the Al-Buraimi region, offering valuable insights into stillbirth risk factors specific to this population. The case-control design enables clear comparisons between stillbirths and live births, while the use of both descriptive statistics and multivariate logistic regression ensures a comprehensive analysis of the data. Additionally, the study includes a large sample size of 128 participants, providing adequate statistical power. Ethical approvals from the Al-Buraimi Hospital Research and Ethics Committee and the Ministry of Health in Oman further enhance the credibility of the study. Moreover, the research is timely and relevant, offering up-to-date data that can inform current clinical practices. 

## Conclusions

This retrospective case-control study identified significant associations between several maternal and foetal factors and stillbirths at Al-Buraimi Hospital in 2024. Maternal factors were associated with stillbirth, while foetal factors included preterm birth, low birth weight, intrauterine growth restriction, and congenital anomalies. Most stillbirths occurred during the antepartum period. Although these findings do not establish causal relationships, they highlight maternal and foetal characteristics that may help identify pregnancies at increased risk of stillbirth. Strengthening antenatal surveillance, optimising the management of maternal comorbidities, and enhancing foetal monitoring may contribute to earlier identification and improved management of high-risk pregnancies. Hospital-based evidence from this study may assist clinicians and policymakers in developing context-specific strategies aimed at improving perinatal care and reducing the burden of stillbirth in the region. Further prospective, multicentre studies are warranted to confirm these associations and better understand the underlying causal pathways.
